# Prognostic signature composed of transcription factors accurately predicts the prognosis of gastric cancer patients

**DOI:** 10.1186/s12935-021-02008-5

**Published:** 2021-07-07

**Authors:** Liqiang Zhou, Zhiqing Chen, You Wu, Hao Lu, Lin Xin

**Affiliations:** 1 Department of General Surgery, The Second Affiliated Hospital of Nanchang University, 1 Minde Road, Donghu District, Nanchang, 330006 Jiangxi China; 2 Molecular Medicine Laboratory, The Second Affiliated Hospital of Nanchang University, Nanchang, 330006 Jiangxi China

**Keywords:** Gastric cancer, Transcription factors, Prognostic signature, Nomogram, ELK3

## Abstract

**Background:**

Transcription factors (TFs) are involved in important molecular biological processes of tumor cells and play an essential role in the occurrence and development of gastric cancer (GC).

**Methods:**

Combined The Cancer Genome Atlas Program and Genotype-Tissue Expression database to extract the expression of TFs in GC, analyzed the differences, and weighted gene co-expression network analysis to extract TFs related to GC. The cohort including the training and validation cohort. Univariate Cox, least absolute contraction and selection operator (LASSO) regression, and multivariate Cox analysis was used for screening hub TFs to construct the prognostic signature in the training cohort. The Kaplan–Meier (K–M) and the receiver operating characteristic curve (ROC) was drawn to evaluate the predictive ability of the prognostic signature. A nomogram combining clinical information and prognostic signatures of TFs was constructed and its prediction accuracy was evaluated through various methods. The target genes of the hub TFs was predicted and enrichment analysis was performed to understand its molecular biological mechanism. Clinical samples and public data of GC was collected to verify its expression and prognosis. 5-Ethynyl-2′-deoxyuridine and Acridine Orange/Ethidium Bromide staining, flow cytometry and Western-Blot detection were used to analyze the effects of hub-TF ELK3 on the proliferation and apoptosis of gastric cancer in vitro.

**Results:**

A total of 511 misaligned TFs were obtained and 200 GC-related TFs were exposed from them. After systematic analysis, a prognostic signature composed of 4 TFs (ZNF300, ELK3, SP6, MEF2B) were constructed. The KM and ROC curves demonstrated the good predictive ability in training, verification, and complete cohort. The areas under the ROC curve are respectively 0.737, 0.705, 0.700. The calibration chart verified that the predictive ability of the nomogram constructed by combining the prognostic signature of TFs and clinical information was accurate, with a C-index of 0.714. Enriching the target genes of hub TFs showed that it plays an vital role in tumor progression, and its expression and prognostic verification were consistent with the previous analysis. Among them, ELK3 was proved in vitro, and downregulation of its expression inhibited the proliferation of gastric cancer cells, induced proliferation, and exerted anti-tumor effects.

**Conclusions:**

The 4-TFs prognostic signature accurately predicted the overall survival of GC, and ELK3 may be potential therapeutic targets for GC

**Supplementary Information:**

The online version contains supplementary material available at 10.1186/s12935-021-02008-5.

## Introduction

Gastric cancer is a common malignant tumor of the digestive tract. Its incidence ranks fifth among all malignant tumors, and its mortality ranks fourth among cancer-related deaths [1]. Due to factors such as economic level and lifestyle, China has become an area with a high incidence of gastric cancer. In 2020, the new cases and deaths of gastric cancer in China accounted for 62.3% and 51.4% of the global total [1]. The clinical manifestations of gastric cancer have no significant specificity, similar to the manifestations of non-malignant gastrointestinal diseases, with insidious onset, rapid progress, difficulty in early diagnosis, and mostly in the middle and late stages of diagnosis, making most cases lose the best opportunity for surgery. The clinical manifestations of advanced gastric cancer are mostly merged by invasion of adjacent tissues or organs, lymph node metastasis in the abdominal cavity and organ metastasis. At present, the main treatment for patients with gastric cancer in this stage is chemotherapy. However, this treatment has large side effects, low quality of life of patients, and short survival period [2]. Therefore, seeking a stable and effective diagnostic index for gastric cancer and solving the problems encountered in clinical treatment is the focus of gastric cancer research.

Transcription factors are a group of protein molecules that can specifically bind to a specific sequence upstream of the 5′end of a gene to ensure that the target gene is expressed at a specific time and space with a specific strength. Their function is to regulate, turn on and turn off genes to guarantee that the correct number of genes expressed in the correct cell at the correct time throughout the entire life of the cell and organism [3]. Among genetic factors, TFs play a vital role in the most important cellular processes, such as cell development, response to internal and external environmental changes, cell cycle control, and carcinogenesis [4]. TFs are the drivers of tumor initiation and disease progression, and their remarkable diversity and effectiveness making their attractive prognostic and therapeutic targets for cancer [5, 6].

In this study, we combined The Cancer Genome Atlas (TCGA) and Genotype-Tissue Expression (GTEx) databases on the gene expression and corresponding clinical information of gastric cancer. Several TFs related to the overall prognosis were identified, and a prognostic signature of TFs was developed to predict the overall survival of gastric cancer patients. Clinical samples and public databases was used to verify the expression and prognosis of these hub TFs. We also jointly established a nomogram with the prognostic signature and clinical information and used a variety of methods to verify its predictive performance, which is helpful for clinicians to make decisions. More importantly, we identified a novel biomarker for gastric cancer and found it regulated cell cycle and proliferation in vitro.

## Materials and methods

### Data processing

The names of transcription factor gene were obtained from the Human Transcription Database website (http://bioinfo.life.hust.edu.cn/HumanTFDB#!/) [7]. Gastric cancer RNA-seq data and clinical information were obtained from the TCGA website. We standardized the downloaded FPKM data of gastric cancer and converted it into TPM data, and combined the TPM data of normal gastric mucosa downloaded on the GTEx website by removing the batch effect. After extracting the transcription factor expression data, using the “limma” R package [8], False Discovery Rate (FDR) < 0.05 and |log2Fold Change (FC)| > 1.0 as screening conditions to identify differentially expressed transcription factors. The “ggplot” R package was used to draw volcano maps and heat maps to visualize differentially expressed transcription factors.

### Weighted gene co-expression network analysis (WGCNA)

To search the TFs that are highly correlated with gastric cancer, the DETFs obtained were analyzed using the “WGCNA” R package [9]. We firstly used the Pearson correlation coefficient of gene pairs to establish an unsupervised co-expression relationship based on the adjacency matrix of connection strength. Then we used topological overlap matrix analysis to cluster the adjacency matrix of the gene expression data of gastric cancer patients. Finally, the dynamic tree-cutting algorithm was applied to the tree diagram for module identification, the minimum size of the module gene number was set to 30, and the cutting height was 0.90. Using the expression data of each co-expression module in all samples to execute module feature genes (MEs) as the first main component. The candidate TFs in the modules with the highest and lowest correlation with GC were extracted for further analysis.

### Construction and verification of prognostic signatures

Univariate Cox regression analysis screened out TFs related to the overall prognosis from DETFs. The LASSO regression was further analyzed and the collinearity was removed to obtain TFs that were significantly related to the prognosis [10]. Then, the samples in TCGA were randomly divided into the training and verification cohort. In the training cohort, multivariate Cox regression was used to construct a prognostic signature in TFs with significant prognosis. The hub TFs with non-zero coefficients were selected to calculate the risk score. The prognostic risk score of each patient applies to the following formula: risk score = expression level of TF 1 × Cof1 + expression level of TF 2 × Cof 2 + … + Expression level of TF x × Cof x, where Cof represents the value of each TF Regression coefficients. The median risk score was used as the cut-off value to divide STAD patients in the training cohort into high-risk and low-risk groups. Using the same formula and the same cut-off value in the verification queue. Drawing the K–M survival curve, and using the log-rank test to evaluate the difference in survival between the high and low-risk groups. The sensitivity and specificity of the prognostic signature was calculated by the 5-year ROC curve [11]. Univariate and multivariate Cox regression analyses were performed to confirm whether the prognostic model of TFs is an independent prognostic factor compared with the clinical prognosis. Also, we merged the training and validation cohorts and used the same methods for analysis.

### Pathway analysis

To explore the molecular mechanism differences between high and low-risk groups. We performed principal component analysis to understand the difference between high and low-risk groups. Using Gene Set Enrichment Analysis (GSEA, version 4.0) based on the molecular signature database (Molecular Signatures Database, MSigDB) to provide gene enrichment analysis for the high-risk group and low-risk group (|NSE| > 1, FDR < 0.05 is considered as statistical learning meaning. Using this method to find the differences in tumor pathways and mechanisms between high and low-risk groups.

### Nomogram construction and verification

Using the “rms” R package to build a prognostic nomogram for STAD patients to predict the probability of survival in 1–5 years. Age, Gender, Radiation therapy, Pharmaceutical therapy, Pathological stage, pathological T stage, pathological N stage, pathological M stage, and risk score are independent parameters that form the nomogram. Using C index and calibration curve to calculate the discrimination and calibration of nomogram prediction and true survival rate [12]. By quantifying the net income under different threshold probabilities in the nomogram, a decision curve analysis was carried out to determine the clinical validity of the nomogram.

### Transcription target gene prediction and enrichment analysis

Using the Gene Transcription Regulation Database (GTRD) database (http://gtrd.biouml.org), we analyzed that within 2000 kb upstream and downstream of the transcription start site, SiteCount ≥ 10 is the target gene bound by hub TFs [13]. The “org.Hs.eg.db” R package was used to perform GO and KEGG function enrichment analysis, among which items that meeting p value < 0.05 and q value < 0.05 are significant, to explore the potential function of hub TF in gastric cancer.

### Hub TFs expression and prognosis characteristics and verification

We first analyzed the relationship between hub TFs and various clinicopathological characteristics, and explored their expression characteristics. Then, 10 pairs of gastric cancer clinical surgical specimens were collected. All procedures were approved by the patient’s informed consent and the ethics committee of the Second Affiliated Hospital of Nanchang University. After the sample was homogenized, the total RNA was extracted with Trizol (Thermo Fisher, USA), and the RNA obtained was reverse transcribed using the reverse transcription kit RR047A (Takara, Japan). ACTB was used as the internal reference gene, and the mRNA expression of hub TFs was analyzed by rt-PCR using the RR820 kit (Takara, Japan) on the 7900-HT system (Thermo Fisher, USA). The primers were all synthesized by Shanghai Shenggong, see the attached table for details. In addition, the HPA database was used to analyze the protein expression of hub TFs [14]. Finally, the prognosis of hub TFs in GSE51105 was verified on Kaplan–Meier Plotter.

### Cell line selection and transfection

Based on the above analysis results, we speculated that ELK3 is a potential new biomarker for gastric cancer. In order to verify the function of ELK3, download the GSE146361 microarray data from Gene Expression Omnibus to analyze the expression of ELK3 in gastric cancer cell lines, and obtain a cell line with high expression of ELK3. Furthermore, according to lipo3000 (Thermo Fisher Scientific, USA) instructions, RNA interference technology was used to inhibit the expression of ELK3 in cells. The siRNA used were all synthesized by Sangon Biotech (Shanghai, China). The sequence is as follows, SiScr: Sense 5′-UUCUCCGAACGUGUCACGUTT-3′, Antisense 5′-ACGUGACACGUUCGGAGAATT-3′; siELK3-1: Sense 5′-CCUGCGAUACUAUUAUGACAATT-3′, Antisense 5′-UUGUCAUAAUAGUAUCGCAGGTT-3′; siELK3-2: Sense 5′-UGGAUCAGAAACAUGAGCAUUTT-3′, Antisense 5′-AAUGCUCAUGUUUCUGAUCCATT-3′; siELK3-3: Sense 5′-AUCAGGUUUGUGACCAAUAAATT-3′, Antisense 5′-UUUAUUGGUCACAAACCUGAUTT-3′. Three days after transfection, the expression changes of ELK3 were analyzed by rt-PCR.

### Cell proliferation assay

The proliferation of gastric cancer cells was evaluated by 5-Ethynyl-2′-deoxyuridine (EDU) Cell proliferation detection. Staining according to the instructions of the EDU commercial kit (US EVERBRIGHT, Suzhou, China), and using a fluorescence microscope (Olympus, Japan) to perform EDU measurement on the treated cells. Performing PI single staining on the cells according to the cell cycle kit (US EVERBRIGHT, Suzhou, China), instructions, using Becton Dickinson FACS calibur instrument to analyze the cell cycle distribution, and analyze the effect of ELK3 on cell proliferation.

### Cell apoptosis detection

In order to analyze the effect of inhibiting ELK3 on the apoptosis of gastric cancer cells, first stained with Acridine Orange/Ethidium Bromide(AO/EB) Kit (Sangon Biotech, Shanghai, China), and analyzed the number of apoptosis of gastric cancer cells after downregulating ELK3. Further, using Annexin V-APC Apoptosis Detection Kit (US EVERBRIGHT, Suzhou, China) to detect cells in the early and late stages of apoptosis. The cells were processed according to the instructions, collected and analyzed in a Becton Dickinson FACS calibur instrument. The cells that were positive for Annexin V-APC and PI were counted.

### Western Blot analysis

The cells were lysed in RIPA (Solarbio, China) containing protease inhibitors (Boster, China) for 20 min on ice. The bicinchoninic acid protein content kit (Solarbio, China) to determine protein concentration. 40 μg total protein per well was separated on 10% polyacrylamide gels and transferred to polyvinylidene fluoride (PVDF) membrane (Merck, Germany). The membrane was blocked with 5% BSA for 1 h at room temperature. The PVDF membrane was combined with GAPDH (Proteintech, USA, Cat No. 60004-1-Ig), PCNA (ABclonal, China, Cat No. A0264), P21 (ABclonal, China, Cat No. A1483), P16 (ABclonal, China, Cat No. A0262), B-cell lymphoma/leukemia-2 (Proteintech, USA, Cat No. 12789-1-AP), BCL2 Associated X (Proteintech, USA, Cat No. 50599-2-Ig), Caspase-3 (Proteintech, USA, Cat No. 66470-2-Ig)was incubated overnight. After washing with Tris-buffered saline Tween, the membrane was probed with horseradish peroxidase-conjugated goat anti-rabbit IgG or goat anti-mouse IgG (Boster, China) for 1 h at room temperature. The band was detected using Super ECL Plus (US EVERBRIGHT, China). The protein expression results are expressed relative to the GAPDH band density.

## Results

### Identification of differentially expressed and gastric cancer-related transcription factors

**Fig. 1:**
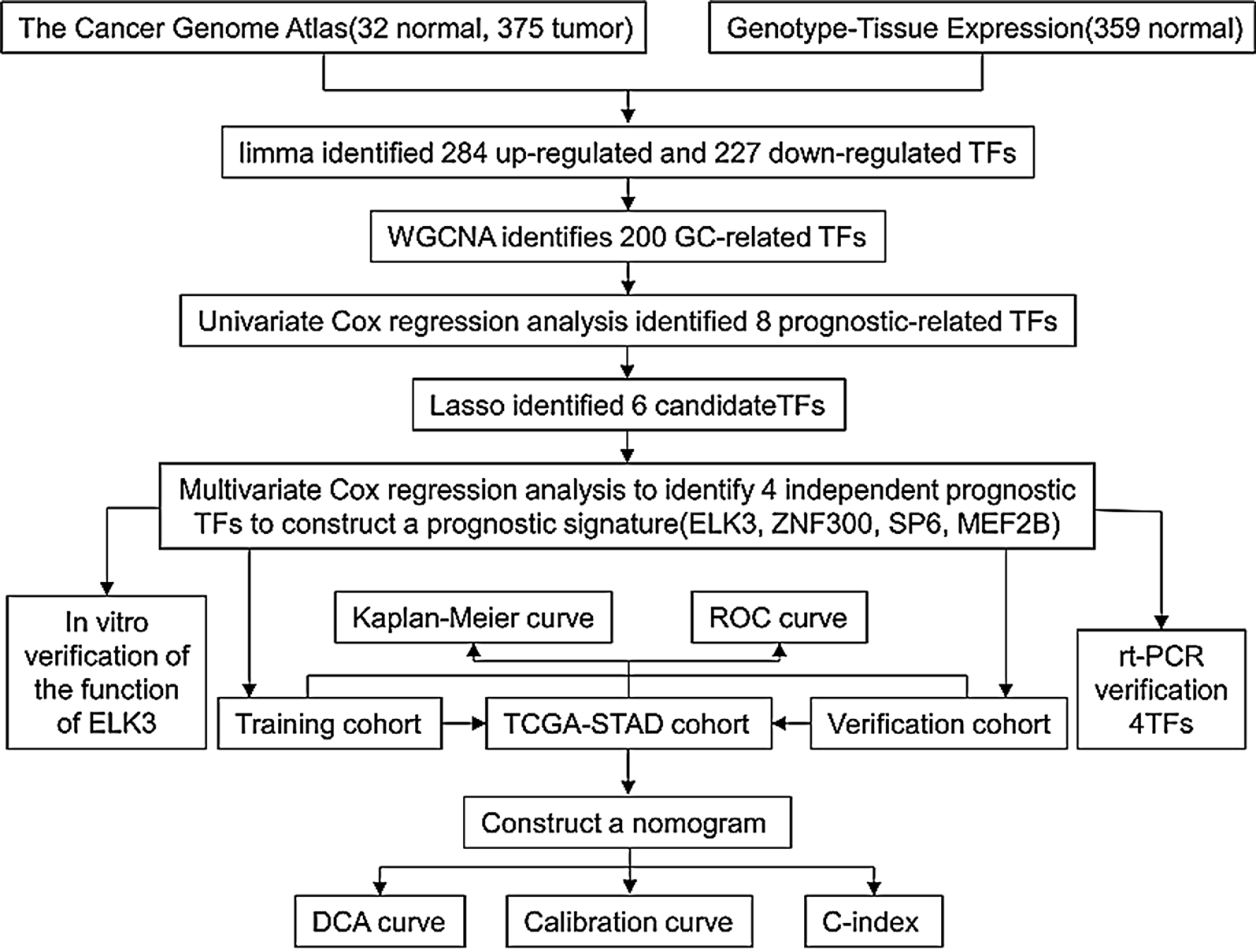
Flow chart of this research

**Fig. 2:**
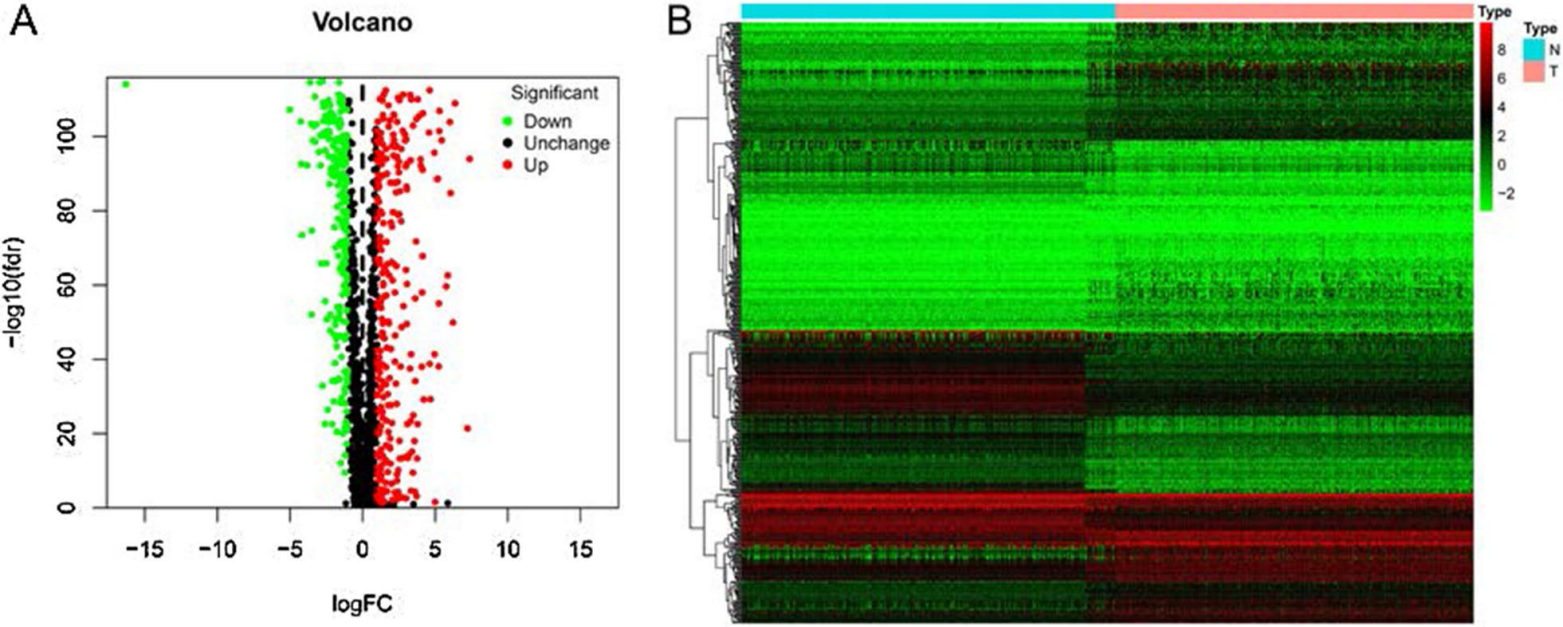
The identifycation of 494 differentially expressed TFs combined with TCGA and GTEx databases. **A** Volcano plot. **B** Heat map

The analysis process of this study was shown in Fig. 1. There are a total of 375 gastric cancer samples and 32 normal samples in the gastric adenocarcinoma (STAD) cohort of the TCGA database, and there are 359 normal samples in the GTEx database. The clinical information of TCGA-STAD was shown in Additional file 1: Table S1. We extracted the expression data of 1,634 transcription factors and identified 284 up-regulated and 227 down-regulated transcription factors based on the screening conditions (Additional file 1: Table S2). Using volcano plot (Fig. 2A) and heat map (Fig. 2B) to visually display.

**Fig. 3:**
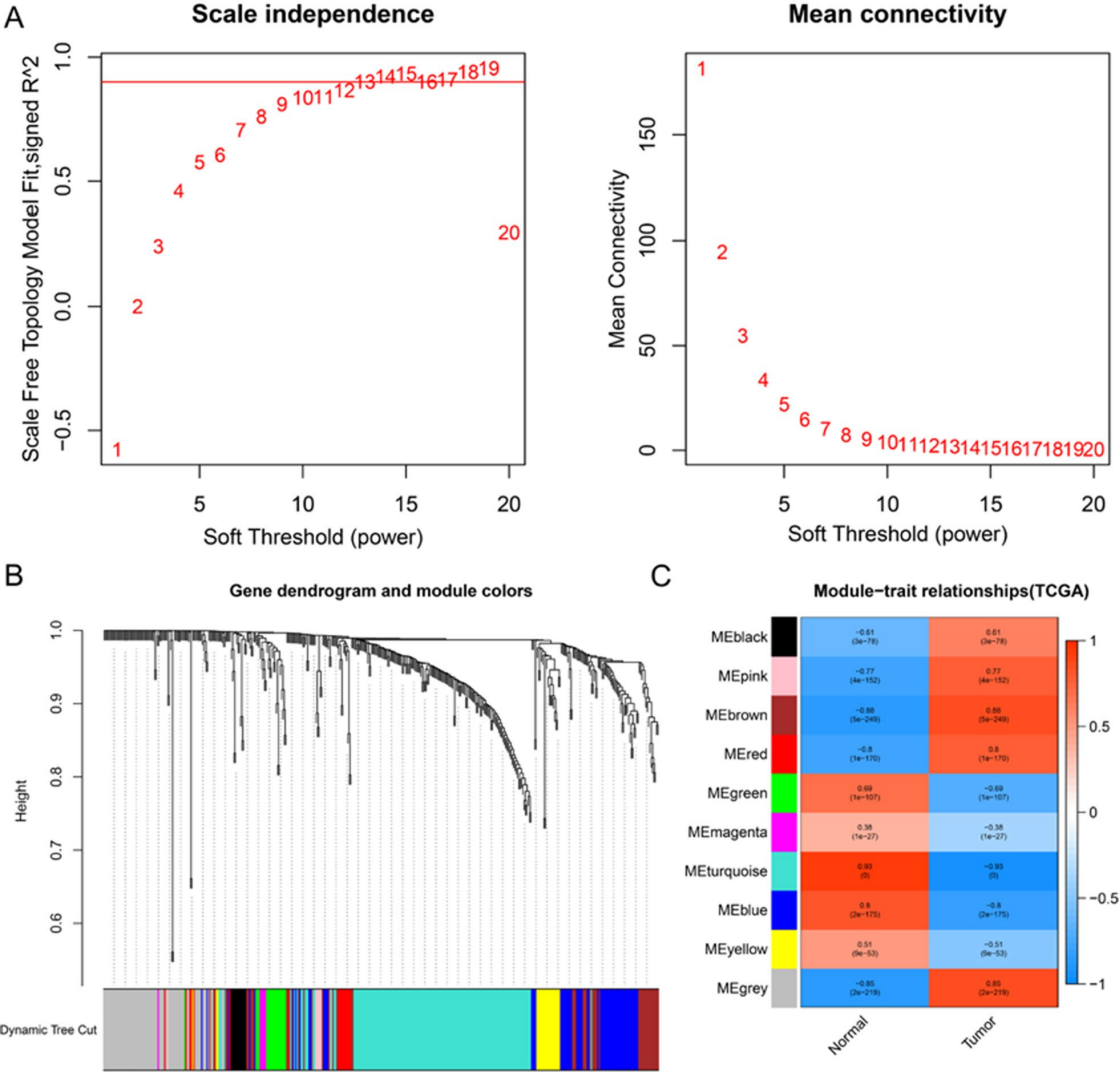
WGCNA identified 200 TFs closely related to gastric cancer. **A** Analyze the best Power value. **B** Dendrogram of all clusters expressing TFs based on the difference metric (1-TOM). **C** The heat map shows the correlation between each module and gastric cancer

Using WGCNA to further analyze these 511 transcription factors. We first determined whether there are outliers in each sample, and then performed hierarchical clustering. In the WGCNA analysis, we chose the soft threshold capability to determine the relative balance of scale independence and mean connectivity. As shown in Fig. 3A, power = 15 can be used as the power value of the soft threshold. Then, based on the input TFs, through average linkage hierarchical clustering, a total of 10 modules were generated (Fig. 3B). After calculating the correlation MS of the shape of each module (Fig. 3C), the MEbrown module containing 49 TFs was considered to be the most relevant to gastric cancer, and the MEturquoise containing 151 TFs was considered the least relevant to gastric cancer. The specific gene names are in Additional file 1: Table S3.

### Construction and verification of TFs prognostic signature

**Fig. 4:**
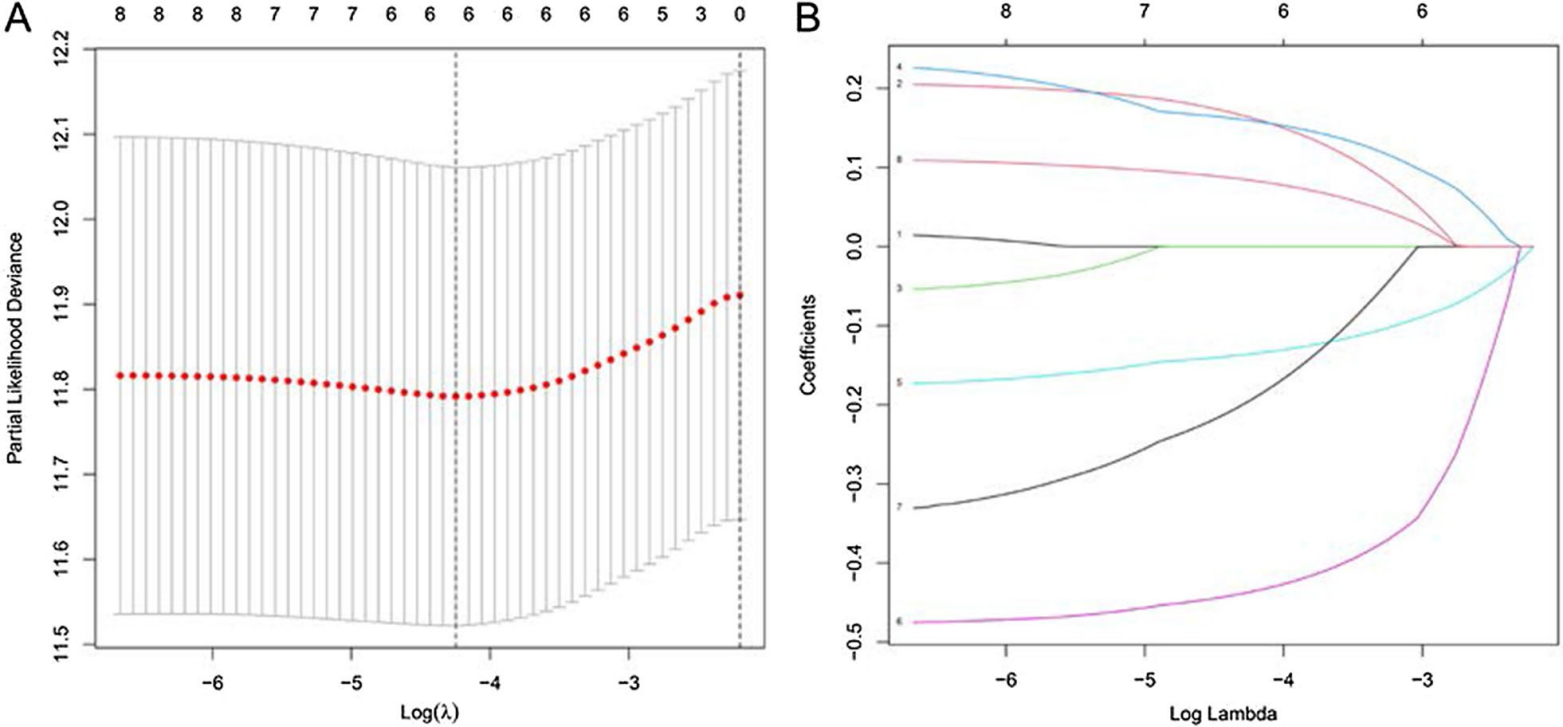
Lasso regression removes collinearity to identify 6 candidate TFs

Univariate Cox regression analysis identified 8 TFs related to the overall prognosis, among which dangerous TFs were shown in red and protective TFs were shown in green (Table 1). The LASSO regression analysis was performed on these TFs to further determine the prognostic significantly related TFs, which are ZNF300, ELK3, SP6, ZNF564, MEF2B, FOXS1 (Fig. 4). Subsequently, we divided the TCGA-STAD queue into a training cohort and a verification cohort. Based on these 4 TFs, multivariate Cox regression analysis was used in the training cohort to further construct the prognostic signature, and finally, 4 TFs were obtained. The relative regression coefficients were shown in Table 1.

**Table 1:** Univariate and multivariate Cox regression screening prognostic-related transcription factors

Gene ID	Univariate Cox regression			Multivariate Cox regression			Coef.
	HR	95% CI	P value	HR	95% CI	P value	
ZNF300	1.248	1.248–1.523	0.030	1.381	1.025–1.860	0.034	0.323
ELK3	1.501	1.177–1.913	0.001	1.371	0.937–2.006	0.104	0.315
SP6	0.802	0.701–0.919	0.001	0.791	0.641–0.977	0.029	− 0.235
MEF2B	0.564	0.340–0.935	0.026	0.503	0.245–1.033	0.061	− 0.686
FOXS1	1.232	1.057–1.437	0.008	–	–	–	–
E2F2	0.796	0.668–0.948	0.011	–	–	–	–
KLF9	1.230	1.061–1.428	0.006	–	–	–	–
ZNF564	0.403	0.230–0.707	0.002	–	–	–	–

*HR* hazard ratio, *CI* confidence interval, *Coef.* coefficients

**Fig. 5:**
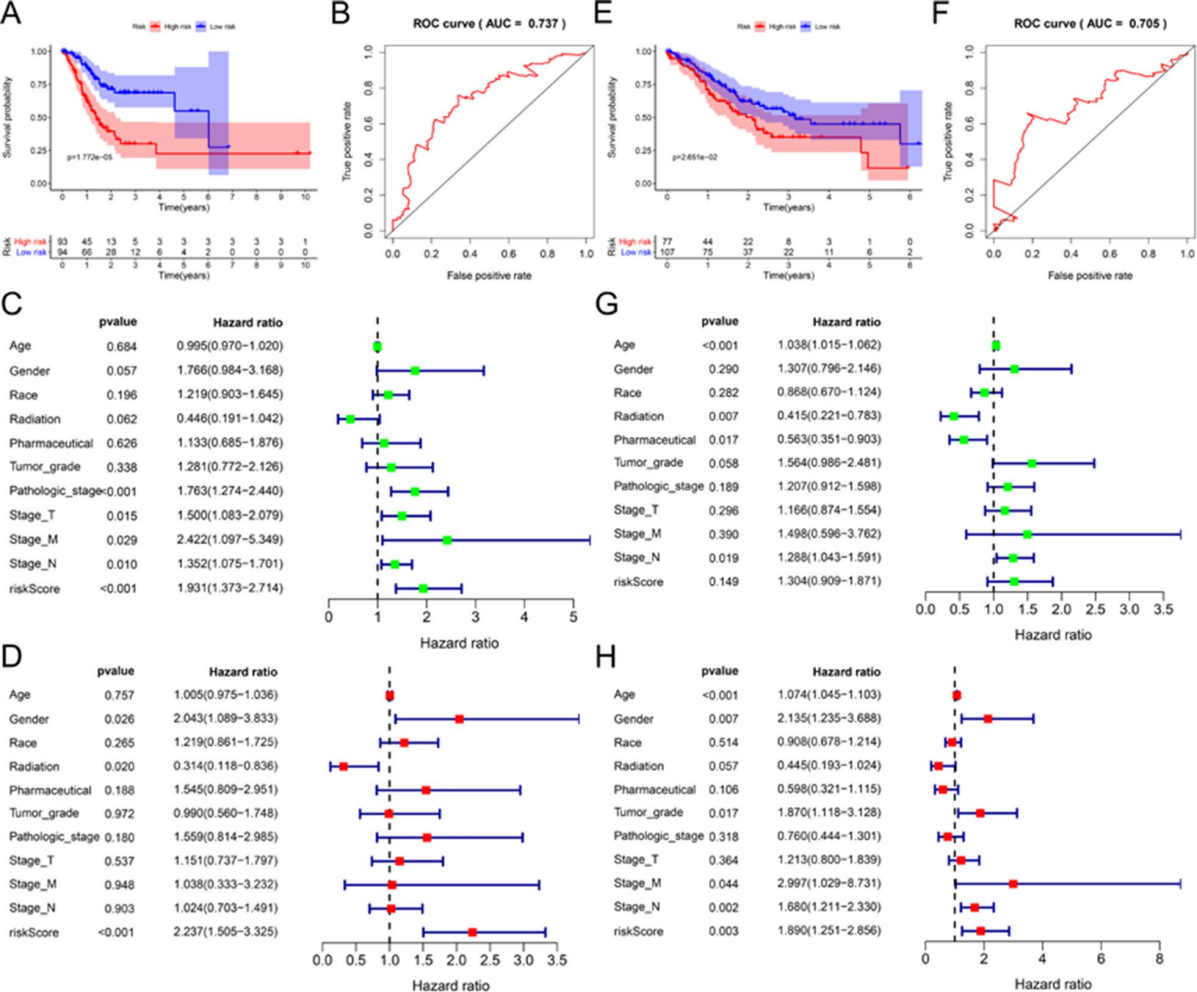
Construction and verification of prognostic signatures in training and verification cohort. **A**, **E** K–M curve analysis of survival differences between high and low-risk groups. **B**, **F** 5-year ROC curve analysis of the sensitivity and specificity of the prognostic signature. **C**, **G** Univariate Cox regression analysis of the relationship between prognosis model, clinical case characteristics, and prognosis. **D**, **H** Multivariate Cox regression analysis whether the prognostic model and clinical case characteristics are independent prognostic factors

By calculating the risk score of each patient, using the median as the threshold, they were divided into high-risk and low-risk groups. Kaplan–Meier (KM) survival analysis showed (Fig. 5A) that the high-risk group had a lower survival rate (P = 1.772e−05). Besides, the 5-year receiver operating characteristic curve (ROC) was drawn and the area under the curve (AUC) was calculated to be 0.737, indicated that the prognostic signature has moderate predictive sensitivity and specificity (Fig. 5B). We performed univariate and multivariate Cox regression analysis to assess the prognostic value of risk scores. Univariate Cox regression showed (Fig. 5C) Pathologic stage [HR = 1.763, 95% CI (1.274–2.440), P < 0.001], T stage [HR = 1.500, 95% CI (1.083–2.079), P = 0.015], M stage [HR = 2.4422, 95% CI (1.097–5.349), P = 0.029], N stage [HR = 1.352, 95% CI (1.075–1.701), P = 0.010] and risk score [HR = 1.931, 95% CI (1.373–2.714), P < 0.001]. Multivariate Cox regression analysis showed (Fig. 5D) Gender [HR = 2.043, 95% CI (1.088–3.833), P = 0.026], Radiation therapy [HR = 0.314, 95% CI (0.314–0.836), P = 0.020], And risk score [HR = 2.237, 95% CI (1.505–3.325), P < 0.001] were independent prognostic factors.

Similarly, we verified the prognostic signature in the verification cohort, and the K–M curve survival analysis showed (Fig. 5E) that the prognosis of the high-risk group was worse (P = 2.651e−02). The 5-year AUC was 0.705, showing good specificity and sensitivity (Fig. 5F). Univariate and multivariate Cox regression analysis showed (Fig. 5G, H), Age [HR = 1.074, 95% CI (1.045–1.103), P < 0.001], Gender [HR = 2.135, 95% CI (1.235–3.688), P = 0.007], Tumor grade [HR = 1.870, 95% CI (1.118–3.128), P = 0.017], M stage [HR = 2.997, 95% CI (1.029–8.731), P = 0.044], N stage [HR = 1.680, 95% CI (1.211–2.330), P = 0.002], risk value [HR = 1.890, 95% CI (1.251–2.856), P = 0.003] are independent prognostic factors.

**Fig. 6:**
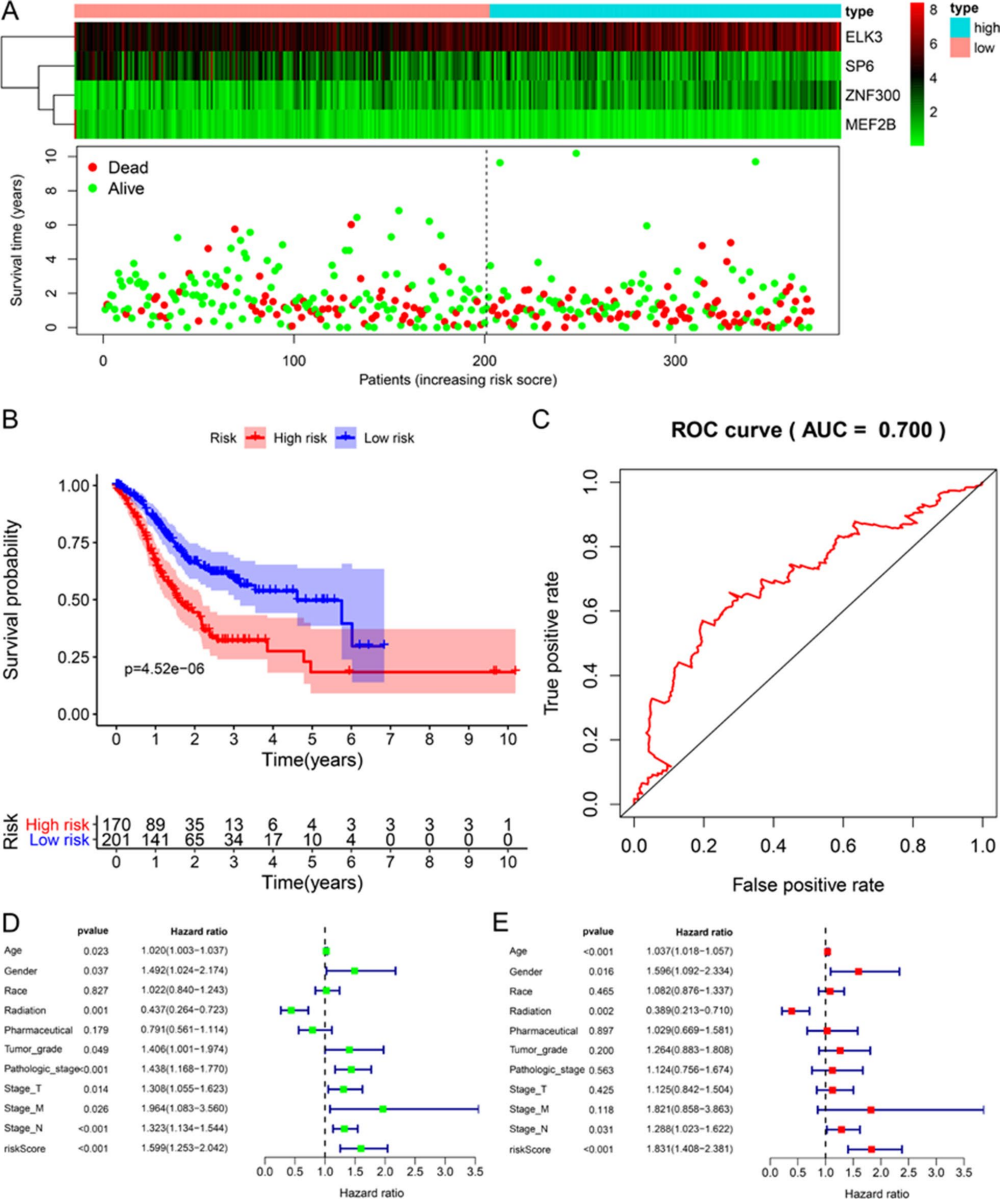
Overall analysis of prognostic signatures in the TCGA-STAD cohort. **A** Heat maps and scatters plots show that the high-risk group has higher mortality and shorter survival time for gastric cancer patients. **B** The K–M curve shows that the high-risk group has a worse prognosis. **C** The 5-year ROC curve shows that the prognostic model has good predictive performance. **D**, **E** Univariate and multivariate Cox regression analysis shows that the prognostic model is an independent prognostic factor

Also, we analyzed the entire TCGA-STAD cohort. The scatter chart showed the distribution of risk scores and the correlation between risk scores and survival data. Patients in the high-risk group had higher mortality and lower survival time (Fig. 6A). The K-M curve survival analysis showed (Fig. 6B) that the low-risk group had a higher survival rate (P = 4.520e−06). The 5-year AUC value is 0.700, which is not significantly different from the training set and the validation set (Fig. 6C). Univariate and multivariate Cox regression analysis showed that compared with Age [HR = 1.037, 95% CI (1.018–1.057), P < 0.001], Gender [HR = 1.596, 95% CI (1.092–2.334)), P = 0.016], Radiation therapy [HR = 0.389, 95% CI (0.213–0.710), P = 0.002], N stage [HR = 1.288, 95% CI (1.023–1.622), P = 0.031] these factors, the risk score [HR = 1.831, 95% CI (1.408–2.381), P < 0.001] has better predictive ability (Fig. 6D, E).

### Pathway analysis

**Fig. 7:**
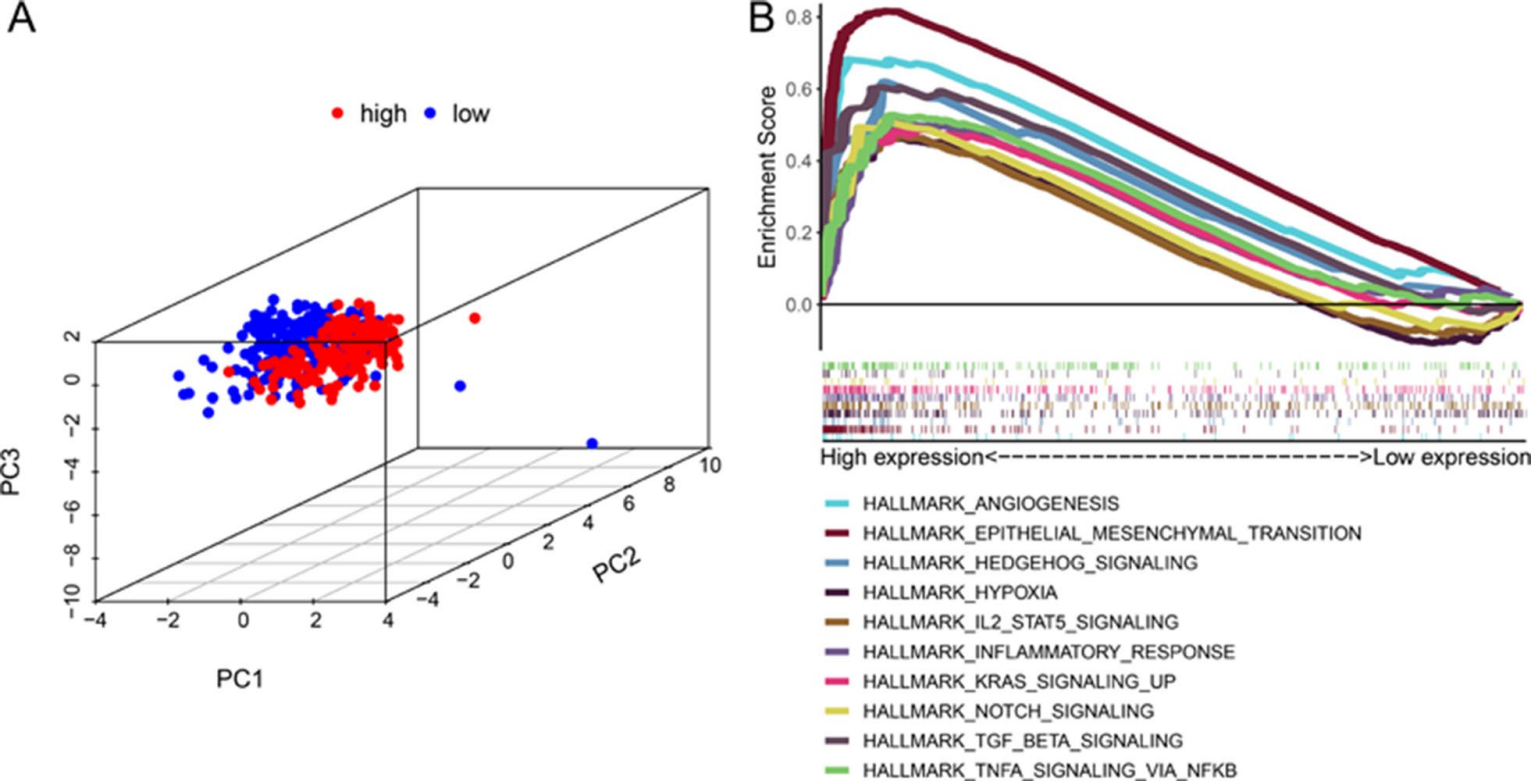
Analyzes the difference between the high and low-risk groups. **A** Principal component analysis. **B** GSEA shows that the high-risk group gastric cancer samples are enriched in pathways closely related to the tumor

First, the principal component analysis (PCA) showed that there were significant differences between the high and low-risk groups (Fig. 7A). Pathway analysis using GSEA (Fig. 7B) showed that gastric cancer samples in the high-risk group were mainly enriched in Angiogenesis, Epithelial Mesenchymal Transition, Hedgehog signaling, Hypoxia, IL2/STAT5 signaling, Inflammatory Response, KRAS signaling up, NOTCH signaling, TGF-BETA signaling, NFKB/TNFA signaling. These pathways play an important role in the occurrence and development of tumors, suggesting that patients with high-risk gastric cancer have a higher degree of tumor malignancy.

### Nomogram construction and verification

**Fig. 8:**
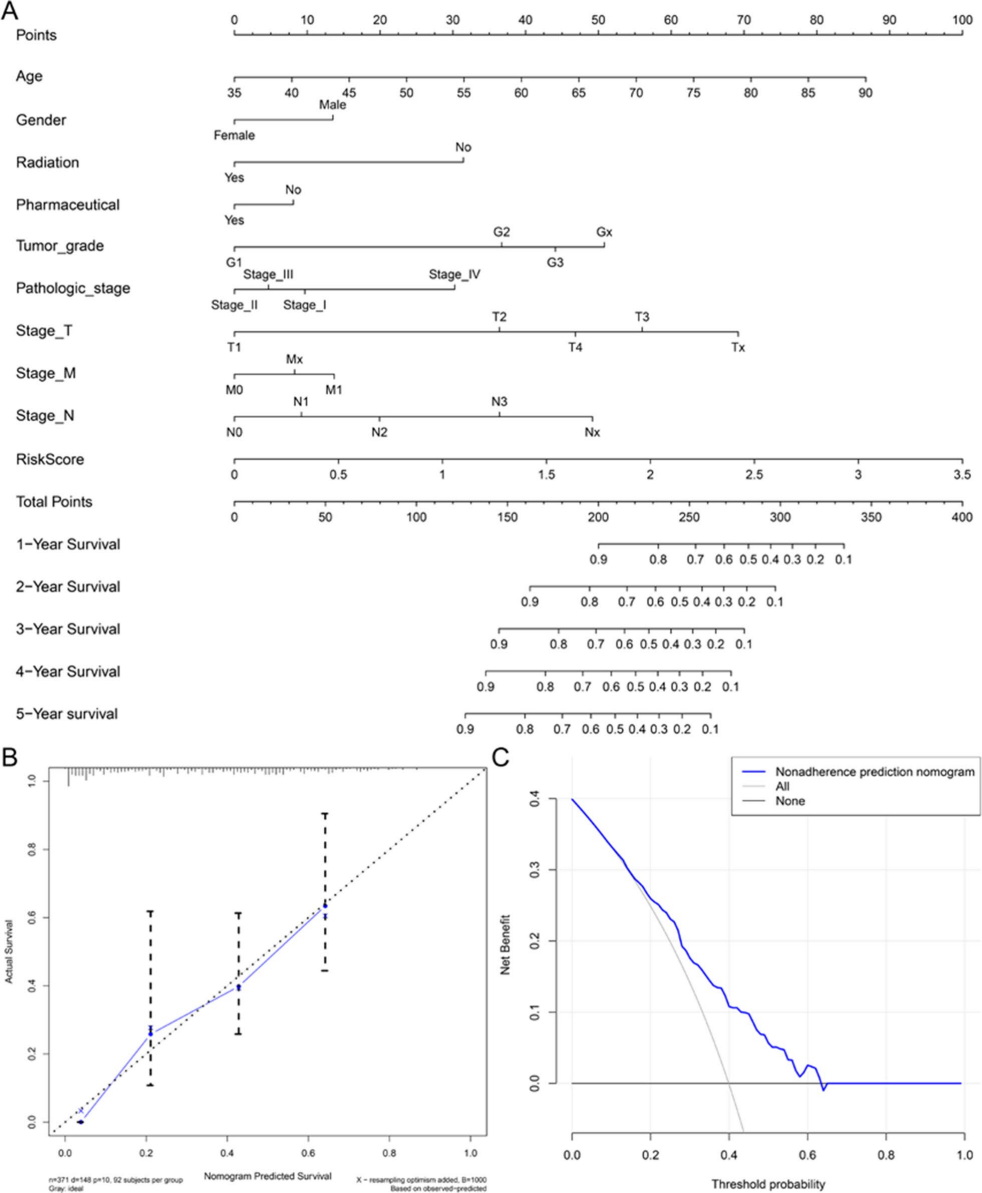
Construction and verification of nomogram. **A** Combining clinicopathological characteristics and prognostic signatures to construct a nomogram to predict the 1–5 year survival rate of gastric cancer patients. **B** The 5-year calibration chart verifies the predictive ability of the nomogram. **C** 5-year decision curve analysis of clinical benefit rate

The nomogram is an effective tool that integrate multiple risk factors for clinical applications. We established a nomogram of the overall prognosis for 1–5 years in the TCGA-STAD cohort. The model integrates Age, Gender, Radiation therapy, Pharmaceutical therapy, Tumor grade, Pathologic stage, T stage, M stage, N stage, RiskScore. The total points of each patient provided the estimated 1–5 year survival times (Fig. 8A). The C-index of this nomogram is 0.714. As shown by the calibration chart, the actual 5-year survival rate matches well with the 5-year survival rate predicted by the calibration chart (Fig. 8B). Decision curve display (Fig. 8C), if the threshold probability of a patient and a doctor is > 14 and < 67%, respectively, using this nomogram to predict gastric cancer patients prognosis more benefit than the scheme. Within this range, the net benefit was comparable with several overlaps, based on the nomogram.

### Hub TFs target gene prediction and enrichment analysis

**Fig. 9:**
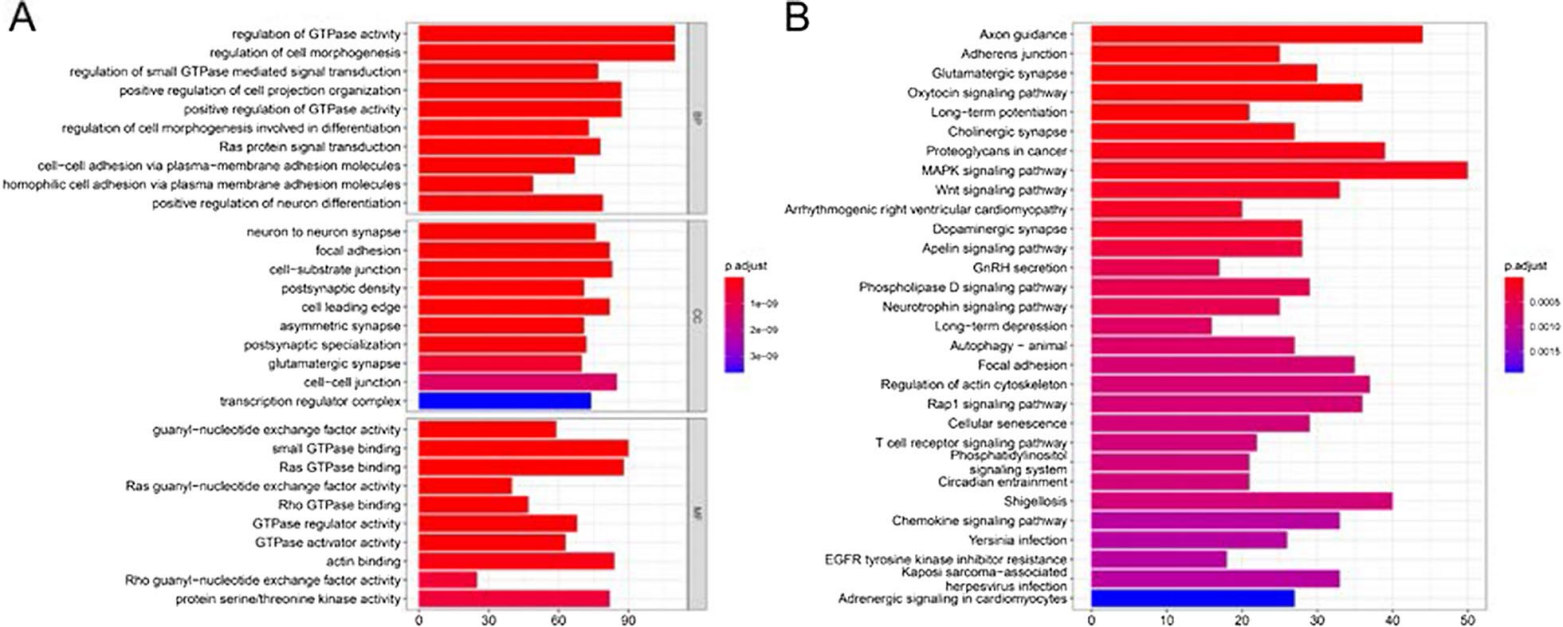
Enrichment analysis of hub TFs target genes. **A** Gene ontology, **B** Kyoto Encyclopedia of Genes and Genomes

The GTRD is used to predict the target genes of 4 hub TFs. Among them, there are 623 eligible target genes for ELK3, 449 for SP6, 1569 for MEF2B, and 89 for ZNF300 (Additional file 1: Table S4). Perform gene ontology (GO) and “Kyoto Encyclopedia of Genes and Genomes” (KEGG) analysis on these target genes. GO showed (Fig. 9A) that biological processes were enriched in regulation of GTPase activity, regulation of cell morphogenesis, Ras protein signal transduction, etc., cell components were enriched in neuron to neuron synapse, focal adhesion, cell-substrate junction, etc., and molecular functions were enriched in guanyl-nucleotide exchange factor activity, small GTPase binding, Ras GTPase binding. For KEGG (Fig. 9B), target genes were mainly enriched in important signals involved in tumorigenesis and development, such as MAPK signaling pathway, Wnt signaling pathway, Autophagy, and Rap1 signaling pathway.

### Hub TFs expression and prognosis characteristics and verification

**Fig. 10:**
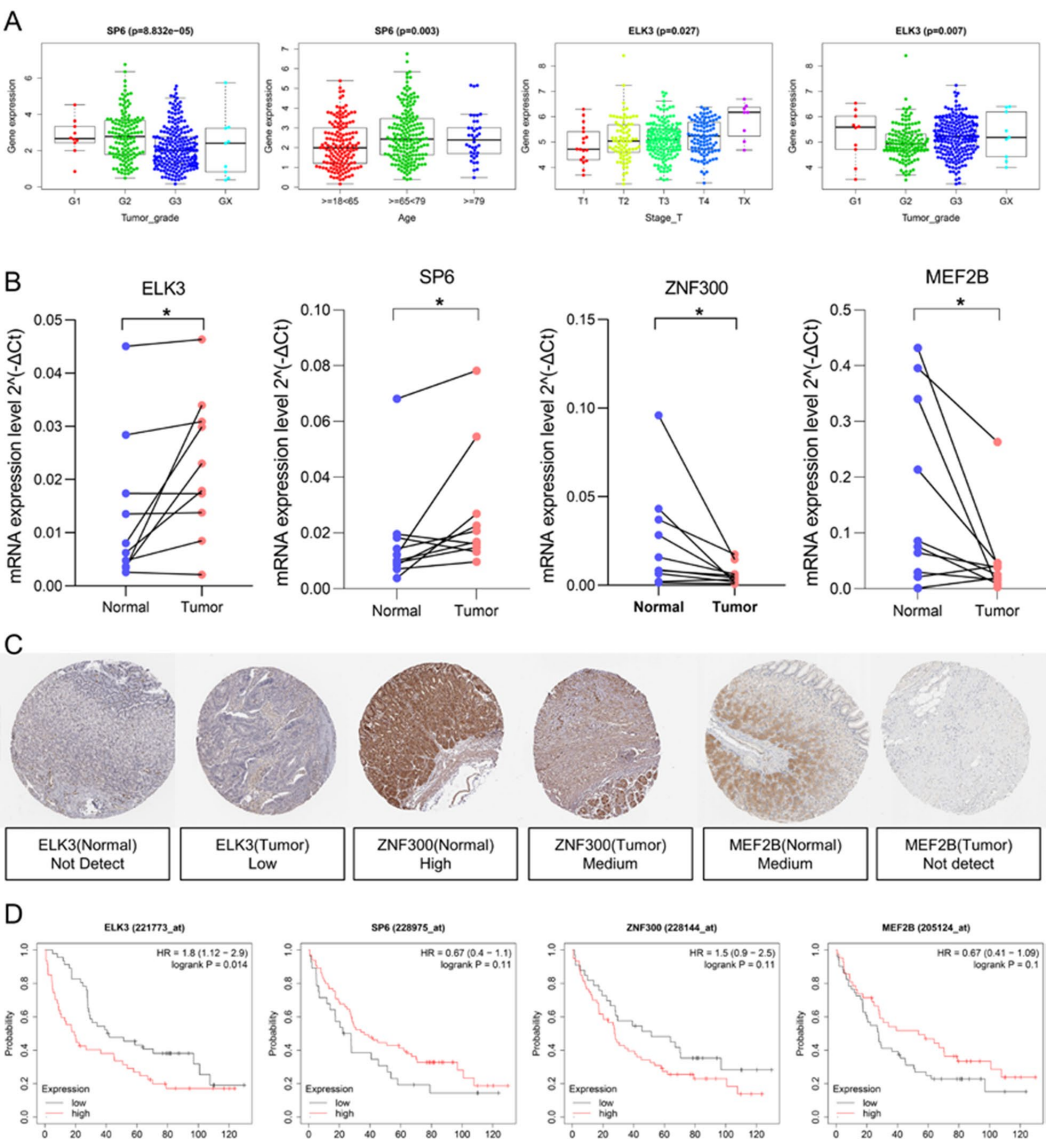
Expression and prognostic verification of hub TFs. **A** rt-PCR analyzes the expression of hub TFs in surgical samples. **B** HPA database analyzes the protein expression level of hub TFs. **C** Analyze the prognosis of hub TFs in GSE51105

We analyzed the relationship between hub TFs and clinicopathological characteristics, and the results showed that the expression of SP6 was related to the grade and age of gastric cancer, and the expression of ELK3 was related to the grade, and its expression increased with the depth of tumor invasion(Fig. 10A). Then, we used rt-PCR to verify the mRNA expression of hub TFs in 10 pairs of clinical samples. The primer sequences are in Table 2. The results suggested (Fig. 10B) that the expression of ELK3 and SP6 is increased in gastric cancer, and the expression of ZNF300 and MEF2B is down-regulated in gastric cancer. In addition, using The Human Protein Atlas (HPA) to analyze the protein expression of hub TFs, ELK3 immunohistochemical staining intensity in normal tissues is lower than gastric cancer tissues, while ZNF300 and MEF2B are higher than gastric cancer tissues (Fig. 10C). Kaplan–Meier Plotter showed that the expressions of ELK3 (P = 0.014) and ZNF300 (P = 0.110) in the GSE51105 data set were associated with poor prognosis, and low expression of SP6 (P = 0.110) and MEF2B (P = 0.100) suggested a better prognosis (Fig. 10D).

**Table 2:** Hub transcription factors primer sequence

Gene ID	Forward primer sequence (5′–3′)	Reverse primer sequence (5′–3′)
ZNF300	GAGTAACCTTCACAACTCCCAG	ATGCCTCAGTCACTGTTTTGC
ELK3	GAGAGTGCA ATCACGCTGTG	GTTCGAGGTCCAGCAGATCAA
SP6	CAGCCTCTCCAAACTTACCAG	AGGTCCTCGCAGGTTACCC
MEF2B	ATGGACCGTGTGCTGCTGAAGT	TCCGAAACTTCTCTCCTGGCTC
ACTB	CACCATTGGCAATGAGCGGTTC	AGGTCTTTGCGGATGTCCACGT

### Inhibition of ELK3 can inhibit the proliferation of gastric cancer cells and induce apoptosis

**Fig. 11:**
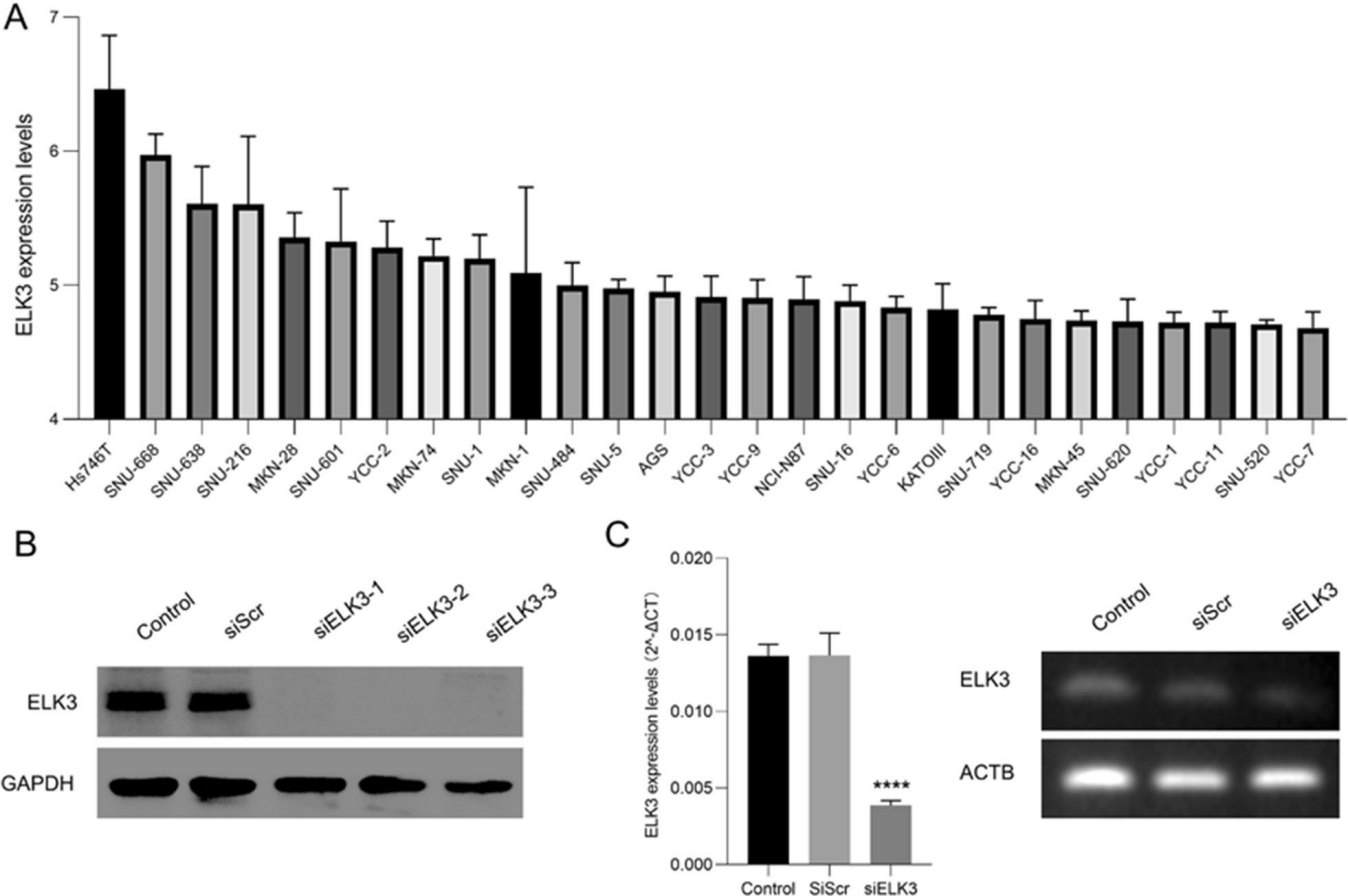
Cell line selection and verification of ELK3 inhibition efficiency. **A** The expression of ELK3 in each gastric cancer cell line in the GSE146361 microarray. **B** The expression of ELK3 in each gastric cancer cell line in the GSE146361 microarray. **C** rt-PCR analysis inhibits the transfection efficiency of ELK3

Based on the above analysis results, we found that ELK3 is not only highly expressed in gastric cancer, but also related to poor prognosis. Therefore, further analysis of the role played by ELK3 in gastric cancer cells. First, we analyzed the expression of ELK3 in 27 cell lines from the GSE146361 microarray, and the results indicated that the expression of Hs746t was the highest (Fig. 11A). Furthermore, using Hs746t as an in vitro verification experimental cell line, three siRNAs were used to inhibit the expression of its ELK3. Western-blot showed that all three siRNAs had good effects (Fig.11B). We chose the second siRNA for further experiments, and rt-PCR showed that it can inhibit the RNA expression of ELK3 in cells (Fig. 11C).

**Fig. 12:**
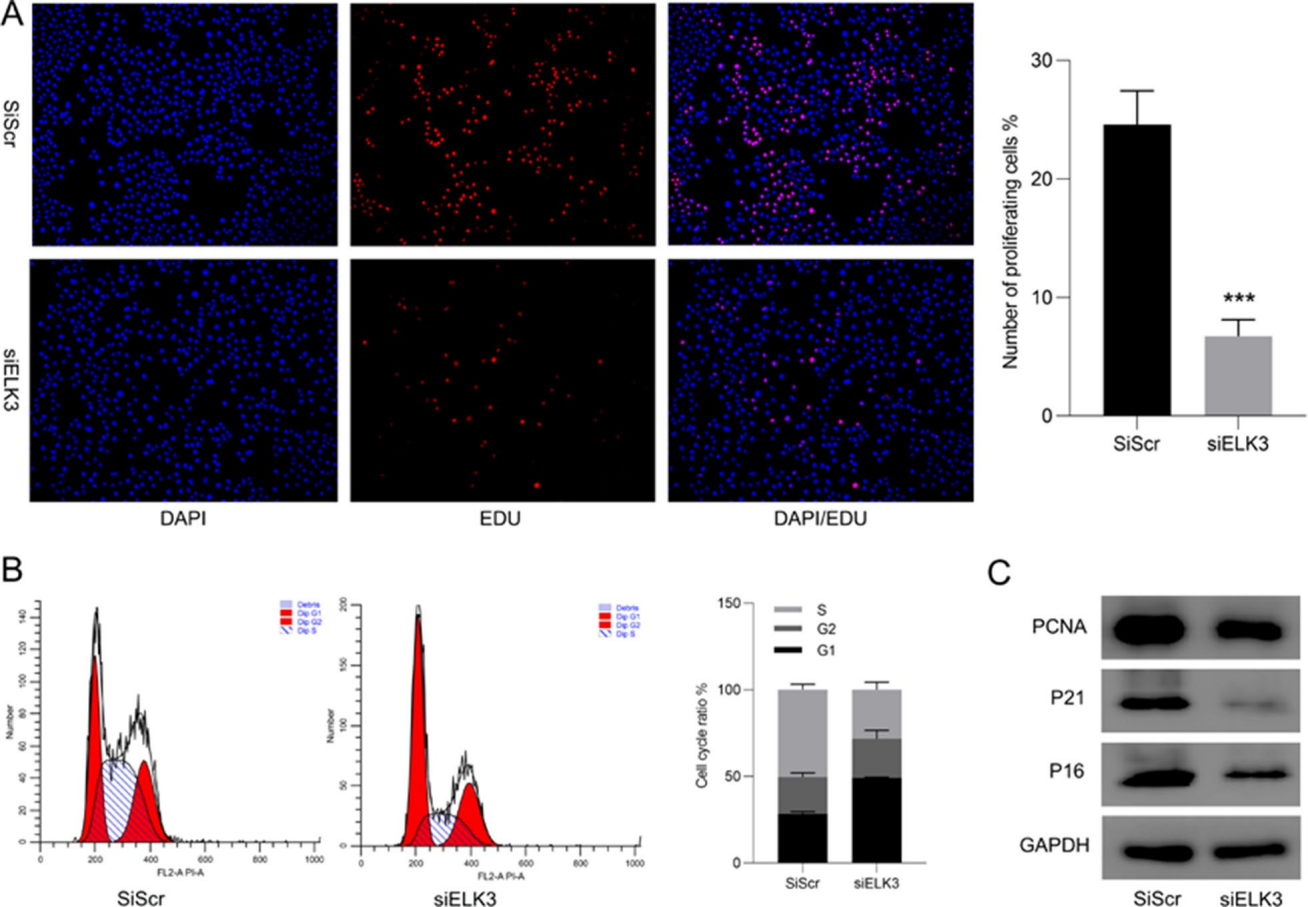
The cell proliferation ability is weakened after ELK3 is inhibited. **A** Inhibition of ELK3 expression cell proliferation decreased. **B** After inhibiting ELK3, gastric cancer cells were blocked in G1 phase. **C** Cell proliferation-related protein after ELK3 expression down-regulation

**Fig. 13:**
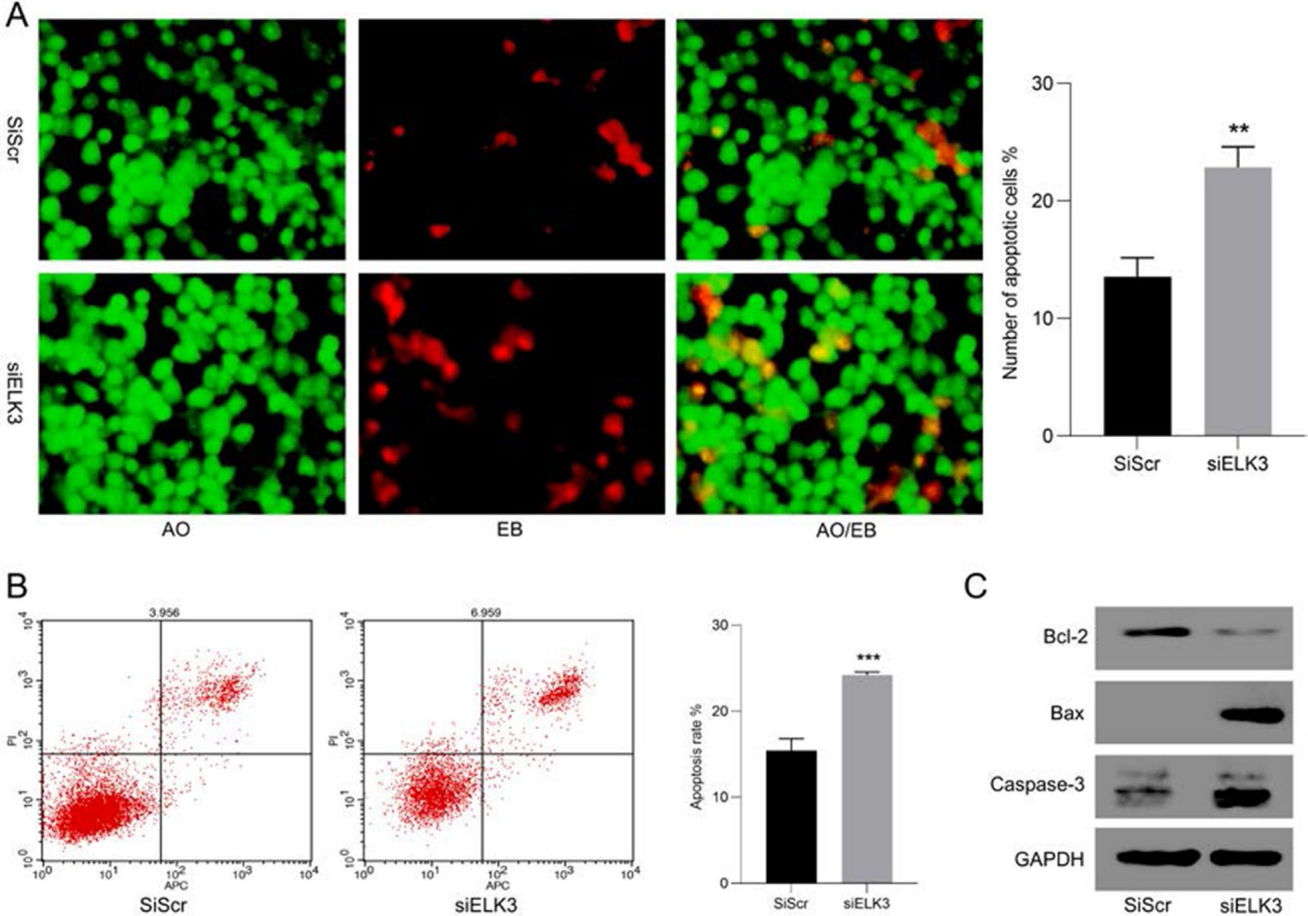
Inhibit ELK3 expression and increase the level of apoptosis in cells. **A** The number of cell apoptosis increased after ELK3 expression was inhibited. **B** Flow cytometry showed that the apoptosis rate of down-regulated ELK3 cells increased. **C** Western-blot showed that the expression of apoptotic protein increased after ELK3 inhibition

In order to verify the effect of ELK3 on the proliferation of gastric cancer, we performed EDU staining. The results showed that the proliferation of Hs746t cells decreased after ELK3 was inhibited (Fig. 12A). Cell cycle analysis indicated that after inhibiting ELK3, the proportion of cells in G1 phase was increased, while that in S phase was decreased, and the cell proliferation ability was weakened (Fig. 12B). Western-blot showed that the expression of cell proliferation-related proteins PCNA, P21, P16 decreased with the down-regulation of ELK3 (Fig. 12C). Finally, we analyzed the effect of inhibiting ELK3 on cell apoptosis. After AO/EB staining, the expression of ELK3 was decreased and the number of apoptosis of Hs746t was increased (Fig. 13A). Flow cytometry detection showed that the rate of apoptosis was negatively correlated with the expression of ELK3﻿ (Fig. 13B). Western-blot showed that after ELK3 was inhibited, the expression of anti-apoptotic protein Bcl-2 decreased, and the expression of pro-apoptotic proteins Bax and Caspase-3 increased (Fig. 13C).

## Discussion

Since the discovery of transcription factors in 1961, there has been increasing evidence that transcription factors are key drivers of many diseases, including cancer [15]. In gastric cancer, there have been multiple reports showing that transcription factors play an important role. For example, cyclic AMP response element binding protein 3-like 4 (CREB3L4) promotes the progression of gastric tumors and endothelial angiogenesis by transcriptionally activating the VEGFA promoter [16]. Insulin gene enhancer protein 1 (ISL1) promotes glycolysis and tumorigenesis in GC through transcriptional regulation of GLUT4 [17]. β-catenin can regulate the expression of PD-L1 to induce immune escape in gastric cancer [18]. These evidence indicated that transcription factors play an important role in gastric cancer. In-depth exploration of the potential molecular functions of transcription factors and using them as therapeutic targets has great prospects.

We jointly analyzed the data of TCGA and GTEx with the differences in the expression of transcription factors in gastric cancer as a whole, and identified transcription factors that are closely related to the prognosis. On this basis, we constructed a prognostic risk proportional model and verified its good predictive performance. Through systematic analysis, we found that the high-risk group has a worse prognosis. GSEA further explained that the high-risk group was mainly enriched in Angiogenesis, Epithelial Mesenchymal Transition (EMT), Hedgehog signaling, Hypoxia, IL2/STAT5 signaling, Inflammatory response, KRAS signaling up, Notch signaling, TGF-beta signaling, NF-κB/TNFA signaling. These signals play an important role in tumors and participate in the occurrence and development of tumors. EMT is one of the key mechanisms of cell morphological plasticity changes in embryonic development and tumor metastasis. It is essential in tumor invasion and metastasis progression. The process was mainly manifested by tumor epithelial cells losing epithelial cell polarity under specific conditions. The contacts between the surrounding cells and the matrix is reduced, the adhesion between the cells is reduced, the interstitial characteristics are obtained, and the cell phenotype is changed. After this process, the tumor cells break through the basement membrane, causing the adhesion between the cells or the matrix to decrease or disappear, migration and invasiveness increase, and enter the lymph and blood vessels to reach distal tissues or organs to form new tumor metastases [19]. Among them, the mechanisms that trigger EMT in tumors include: transforming growth factor-β (TGF-β), Wnt, Notch, and Hedgehog signals. This indicated that the high-risk group of gastric cancer patients progresses more rapidly and has a higher degree of malignancy [20].

We also identified 4 hub TFs, MEF2B, SP6, ZNF300, ELK3, and predicted their target genes. The enrichment analysis of these target genes showed that the target genes were mainly enriched in important signals involved in tumorigenesis and development, such as MAPK signaling pathway, Wnt signaling pathway, Autophagy, and Rap1 signaling pathway. MEF2B is a member of the MEF2 family of proteins and is a transcription factor involved in the development of muscles, heart, bones, blood vessels, and the immune system. However, studies have found that MEF2B can activate the β-catenin pathway to induce lung cancer cell invasion [21]. This may be due to differences in gene expression and functions in different microenvironments. SP6 belongs to the transcription factor family, which contains three classic zinc finger DNA binding domains, which are composed of two cysteines and two histidines (C_2_H_2_ motif) tetrahedral coordinated zinc atoms, these transcription factors bind to GC-rich sequences and related GT and CACCC boxes [22]. At present, there is no experimental study on the mechanism of SP6 in affecting tumor progression. In this study, MEF2B and SP6 were considered protective genes, while ELK3 and ZNF300 were considered oncogenes. ZNF300 is a novel KRAB/C_2_H_2_ gene encoding 68kD ZFP, and its KRAB domain exhibits transcriptional repressive activity [23]. What is interesting is that Endogenous ZNF300 binds directly to the IL2RB gene promoter and potentially activates its expression [24]. Reports in tumors indicated that ZNF300 can promote the progression of cancer cells by activating NF-κB and MAPK pathways to induce tumor cell proliferation, invasion, and drug resistance [25, 26].

It is worth noting that although ZNF300 is associated with poor prognosis, it is low expressed in tumors. The expression of ELK3 in gastric cancer is positively correlated with poor prognosis. ELK3 (also known as Net, SAP-2, or ERP) is a member of the ETS transcription factor family and is located on chromosome 12q23.1. The ELK3 protein often forms a ternary complex transcription factor together with serum response accessory protein 1, which can bind to a specific DNA sequence rich in purine GGA core sequences and regulate the expression of a variety of genes including proto-oncogenes [27]. Under basic conditions, ELK3 is a transcriptional repressor, but it can be activated by RAS/ERK signals and mitogen-activated protein kinase (Mitogen-Activated Protein Kinase, MAPK) pathways to turn it into a transcription activator [28, 29]. In recent years, ELK3 has been proven to play an important role in the occurrence and development of breast cancer, liver cancer, lung cancer, and other malignant tumors [30–33]. In prostate cancer studies, it has been shown that inhibition of ELK3 can promote cycle arrest and apoptosis of tumor cells [34]. In the reports of breast cancer and colorectal cancer, ELK3 is closely related to chemotherapy resistance, and down-regulating its expression can promote chemotherapy sensitivity [35, 36].In addition, ELK3 is also involved in TGF-β signaling to promote tumor cells to undergo epithelial-mesenchymal transition [37, 38]. In gastric cancer, ELK3 has no experimental studies to confirm its function. Our in vitro studies have shown that inhibiting the expression of ELK3 in gastric cancer cell lines reduces its proliferation ability and increases its apoptosis level. The above evidence suggested that ELK3 may act as an oncogene in gastric cancer, but its specific mechanism affecting the progression of gastric cancer requires further experimental research.

## Conclusions

In general, we analyzed the expression differences of transcription factors in gastric cancer based on public databases, screened transcription factors with prognostic ability, and used clinical samples for expression verification. On this basis, we also constructed a prognostic signature and nomogram and systematically verified that it has good predictive sensitivity, which is helpful for accurate and personalized treatment of gastric cancer. More importantly, we have identified ELK3 as a new biomarker for gastric cancer, which is beneficial to the precise treatment of gastric cancer.

## Supplementary Information

**Additional file 1: Table S1.** Clinical information of the TCGA-STAD cohort. **Table S2.** Differentially expressed transcription factors in the TCGA-STAD cohort. **Table S3.** Transcription factors in modules related to gastric cancer. **Table S4.** predicts target genes of hub TFs.

## Data Availability

All data can be obtained from the corresponding author’s office and public databases.
